# Evolution of a Complex Locus: Exon Gain, Loss and Divergence at the Gr39a Locus in Drosophila

**DOI:** 10.1371/journal.pone.0001513

**Published:** 2008-01-30

**Authors:** Anastasia Gardiner, Daniel Barker, Roger K. Butlin, William C. Jordan, Michael G. Ritchie

**Affiliations:** 1 School of Biology, University of St. Andrews, St. Andrews, Scotland, United Kingdom; 2 Department of Animal and Plant Sciences, University of Sheffield, Sheffield, United Kingdom; 3 Institute of Zoology, Zoological Society of London, London, United Kingdom; Wellcome Trust Centre for Human Genetics, United Kingdom

## Abstract

**Background:**

Gene families typically evolve by gene duplication followed by the adoption of new or altered gene functions. A different way to evolve new but related functions is alternative splicing of existing exons of a complex gene. The chemosensory gene families of animals are characterised by numerous loci of related function. Alternative splicing has only rarely been reported in chemosensory loci, for example in 5 out of around 120 loci in *Drosophila melanogaster*. The gustatory receptor gene *Gr39a* has four large exons that are alternatively spliced with three small conserved exons. Recently the genome sequences of eleven additional species of Drosophila have become available allowing us to examine variation in the structure of the *Gr39a* locus across a wide phylogenetic range of fly species.

**Methodology/Principal Findings:**

We describe a fifth exon and show that the locus has a complex evolutionary history with several duplications, pseudogenisations and losses of exons. PAML analyses suggested that the whole gene has a history of purifying selection, although this was less strong in exons which underwent duplication.

**Conclusions/Significance:**

Estimates of functional divergence between exons were similar in magnitude to functional divergence between duplicated genes, suggesting that exon divergence is broadly equivalent to gene duplication.

## Introduction

Gustatory receptor (*Gr*) genes comprise a large fraction (∼50%) of the Drosophila chemosensory receptor gene superfamily [1], encoding 7-transmembrane (7TM) proteins involved in taste and smell. Most Drosophila *Grs* are very divergent, sometimes showing as little as 8% amino acid identity to each other [1]. Much of the diversity of the chemoreceptor family has evolved through widespread and repeated whole-gene duplications, followed by functional divergence of those duplicates that do not degrade to pseudogenes [2], [3]. Another mechanism that enlarges the eukaryotic protein repertoire in general is alternative splicing. Although this is currently thought to be rare among chemosensory receptor loci [4], [5], three *D. melanogaster Gr* genes (*Gr23a, Gr28b*, *Gr39a*) are notable in that they have been shown to undergo alternative splicing, together coding for 11 proteins, or 16% of all gustatory receptors in the species [1], [6]. Analysis of the *Gr* repertoire in 12 Drosophila species allowed us to identify the orthologues of the *Gr39a* genes in these species [7]. This locus showed an unusual pattern of structural changes compared with other *Grs*. Here we investigate in detail the evolution of the *Gr39a* gene using a comparative, bioinformatic approach.

Located on the left arm of the second chromosome of *D. melanogaster*, the *Gr39a* gene has four large exons (*A*, *B*, *C* and *D*), each including coding sequences for six transmembrane domains, followed by three small exons that together encode the seventh transmembrane domain and COOH-terminus [6]. Any one of the large exons may be spliced to the smaller exons, generating four different 7TM protein products. These are expressed in the main taste organs of *D. melanogaster*, the labellum (Gr39aA, Gr39aB, Gr39aC, Gr39aD), though some are also expressed in the thorax (Gr39aC, Gr39aD), abdomen (Gr39aC) and wings (Gr39aD) [6]. The function of *Gr39a* is unknown, but its close phylogenetic affinity with the *D. melanogaster* male specific pheromone receptor *Gr68a* [8] suggests a possible involvement in pheromone recognition [9].

**Figure 1:**
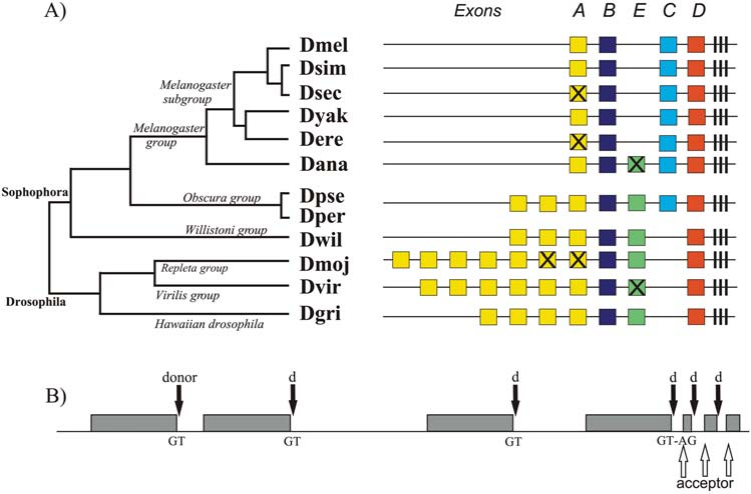
Structure of the *Gr39a* locus and splicing pattern with species' phylogeny. A. Schematic presentation of the *Gr39a* structure (in order) in *D. melanogaster, D. simulans, D. sechellia, D. yakuba, D. erecta, D. ananassae, D. pseudoobscura, D. persimilis, D. willistoni, D. mojavensis, D. virilis, D. grimshawi.* Degraded exons or exons accumulated nonsense mutations are crossed. B. Schematic of the pattern of alternative splicing in the *Gr39a*. Large exons have the 5′-donor site (GT), while the first 3′-acceptor site (AG) appears in the beginning of the block of three conserved small exons.

We have annotated the orthologs of *D. melanogaster Gr39a* in eleven other recently sequenced Drosophila species [10], representing a wide range of phylogenetic divergence from *D. melanogaster* (Figure 1A, [10]). Of these species, nine (*D. melanogaster, D. simulans, D. sechellia, D. yakuba*, *D. erecta*, *D. ananassae*, *D. pseudoobscura*, *D. persimilis* and *D. willistoni*) are in the subgenus *Sophophora*, and the remaining three (*D. mojavensis*, *D. virilis* and *D. grimshawi*) are within the subgenus *Drosophila*. The two subgenera are estimated to have diverged from each other 40–60 million years ago [11], [12]. Lower level groups and subgroups have been identified within the subgenera (Figure 1A). We examined the structural and potentially functional differences between the *Gr39a* genes across these twelve species. We identified a new large exon, exon *E*, found in most species but lost in the melanogaster lineage. After an analysis in which we employ phylogenomic approaches more usually used to examine gene evolution, we propose a model of the evolution of *Gr39a*. We conclude that, despite strong purifying constraints on the *Gr39a* locus overall, the exons that are prone to duplication or pseudogenisation show evidence of relaxed selection which probably facilitated “subfunctionalization” of the duplicated exon copies. Evidence of positive selection also suggests “specialization” and/or neofunctionalization of tandemly duplicated exons has occurred, though potential new functions are currently unknown.

## Results

Chemosensory receptor repertoires among different insects show great divergence, for instance only a few orthologous groups were identified when the complete olfactory and gustatory receptor repertoires of the fruit fly, honeybee and mosquito were compared [4], [5], [13]. Robertson and Wanner [5] suggest that the exons *Gr39aA-D* of *Drosophila melanogaster* form an orthologous group with seventeen *Gr* genes of *Anopheles gambiae* (*AgGr9a*-*n*, *AgGr10*, *AgGr11*, and *AgGr12*), but the evidence of the orthology is lacking due to the very weak bootstrap support for this clade. In *Apis mellifera*, the orthologs of *Gr39a* were not identified [5]. The *Drosophila* exons are related among themselves by duplications, which seem to have occurred after the *Drosophila*-*Anopheles* split.

We were able to uncover complex structural changes that have occurred to *Gr39a* since the subgenera *Drosophila* and *Sophophora* diverged. The gene structure of *Gr39a* described for *D. melanogaster,* with four large exons *A, B, C,* and *D* followed by three small constitutive exons [6], is peculiar to the species of the melanogaster subgroup only (Figure 1A). In this subgroup, the first exon *A* has either accumulated frame shift mutations or significantly degraded in two species: *D. sechellia* and *D. erecta*, respectively. Each large exon contains its own start codon and 5′ splice signal allowing the locus to encode several protein products independent of the mutational alteration of the ORF in one of the exons, so we would expect the exons *B, C* and *D* to be expressed in *D. sechellia* and *D. erecta*. Species of the melanogaster subgroup have also lost an ancestral exon *E,* first described here and identified as a degraded copy in *D. ananassae*, an intact exon in the species of the obscura group, *D. willistoni, D. mojavensis* and *D. grimshawi*, and an exon with two frame-shift mutations in *D. virilis* (Figure 1A). In support of this interpretation, a search for evidence of exon *E* in the melanogaster subgroup revealed the presence of short sequences that code for about 100 amino acids alignable with the truncated exon *E* of *D. ananassae* and intact exon *E* of *D. pseudoobscura*.

**Figure 2:**
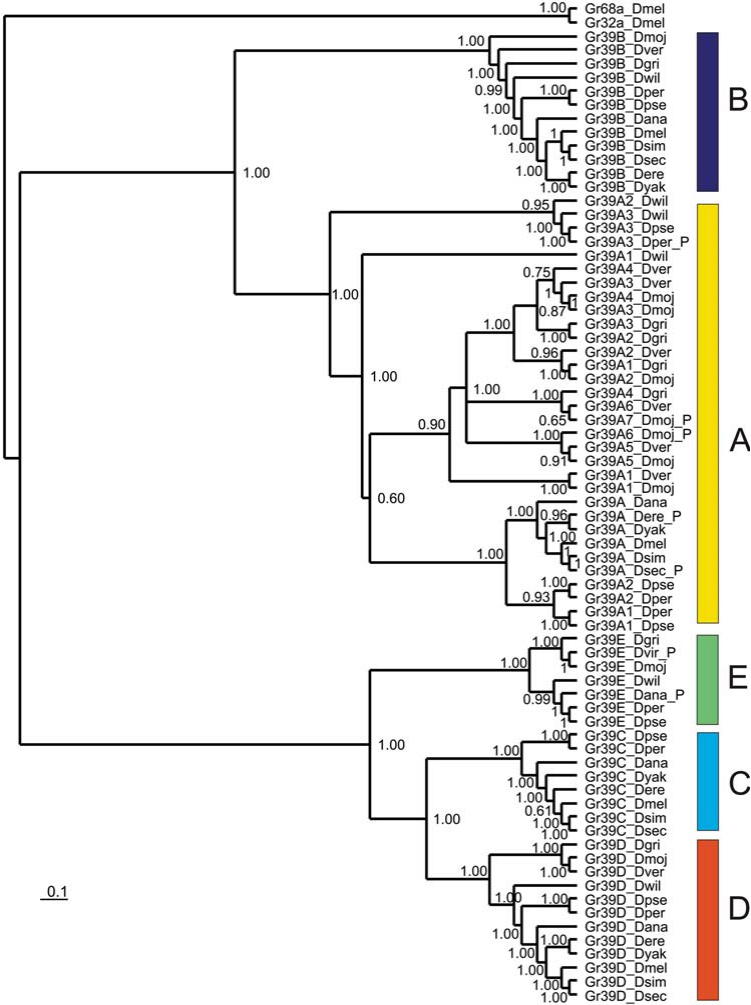
*Gr39a* “exons” tree for 12 *Drosophila* species. Species: Dmel (*D. melanogaster*), Dsec (*D. sechellia*), Dsim (*D. simulans*), Dyak (*D. yakuba*), Dere (*D. erecta*), Dana (*D. ananassae*), Dpse (*D. pseudoobscura*), Dwil (*D. willistoni*), Dmoj (*D. mojavensis*), Dvir (*D. virilis*), Dgri (*D. grimshawi*). Exons with nonsense mutations are indicated by symbol “P”. Numbers show clade posterior probabilities.

The Bayesian-MCMC phylogeny of *GR39a* exons and their homologues is summarized in Figure 2. The posterior probabilities shown in Figure 2 appear to lack significant bias as estimates of the probabilities of the clade, conditional on the data, model and priors: average standard deviation of split frequencies was 0.0060 at the start of the final MCMC sample (close to the ideal value of zero), and lag-1 ACF for lnL in the two MrBayes runs was 0.014 (P>0.05) and 0.036 (P>0.05).

**Figure 3:**
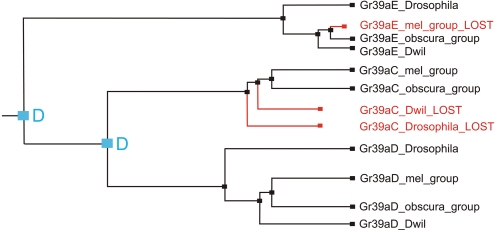
Predictions of duplication (in blue) and loss (in red) events by parsimony.

Exon *E* occupies a basal position within a clade including exons *C* and *D* (Figure 2). The reconciliation of the species tree and phylogenetic tree of exons *E, C* and *D* by the Notung program [14] showed two events of exon duplication (duplication of exon *E* and subsequent origin of exons *D* and *C* before the subgenera *Sophophora* and *Drosophila* split) and three independent events of exon loss (Figure 3). This prediction supports the loss of exon *E* in the melanogaster group, and suggests the loss of exon *C* in *D. willistoni* and the subgenus *Drosophila*, but we did not find any evidence of the presence of exon *C* in these species.

Closely related to each other, exons *B* and *A* were found in all species, *B* as a single copy exon, while exon *A* underwent multiple duplications in several lineages (Figure 1A). The relationships among copies of exon *A* are complex. We found three copies of exon *A* in the obscura and willistoni groups, seven copies (two pseudo- and five intact exons) in *D. mojavensis*, six intact copies in *D. virilis* and four in *D. grimshawii* (Figure 1A and Figure 2). Some exon copies are very recent and/or experienced gene conversion, others are likely to be of more ancient origin, possibly suggesting losses as well as gains. Such extensive exon duplication in an alternatively spliced chemoreceptor gene is an apparently unique case-analyses of the other alternatively spliced gustatory (*Gr23a, Gr28b*) and olfactory (*Or46a, Or69a*) receptor genes revealed a more conservative structure. Thus, the structure of *Gr23a,* with two large alternatively spliced exons, is preserved in all species examined. We found only cases of species-specific degradation or loss of some exons in the *Gr28b, Or46a* and *Or69a* genes due to accumulation of frame shifts, premature stop codons and large deletions (for example, *Gr28bA* in *D. sechellia*, *Gr28bD* and *E* in *D. grimshawii, Or69aC* in *D. sechellia* and *D. melanogaster*).

The presence of sequence motifs that specify six transmembrane domains was detected in all the large exons of *Gr39a*; the last three small exons are present in all species and encode for the seventh transmembrane domain (Figure 1A). We analysed the splicing structure of *Gr39a* in all species. The location of splice sites is conserved throughout the genes examined (Figure 1B). All large exons (except the pseudo-exons *A* in *D. sechellia* and *D. erecta*) and two conserved small exons contain the 5′ donor-splice motif [AG]↑**GT**(NNGT), while the first 3′ acceptor-splice motif (C_n_T_n_)NC**AG**↑[GC] appears at the beginning of the block of three conserved small exons (Figure 1B). This structure supports a model of mutually exclusive alternative splicing, when a single large exon is spliced with the small conserved exons and the other large exons are excluded as part of an intron.

The estimated *ω* values for the whole gene and its large exons were all substantially lower than 1 (Table 1) suggesting that all parts of the gene are subject to strong purifying selection, however weaker selective constraints act on exon *E* and the duplicated exon *A* (*ωE* = 0.27 and *ωA* = 0.24; c.f. ∼0.17).

**Table 1:** PAML analysis of selection on the *Gr39a* gene and its tandemly duplicated large exons (*A, B, C, D, E*).

Region	*N*	*ω*M0	Test for positive selection		
			Site models *M7 vs. M8*	Branch-site models *M1* vs. *M2*	Branch models for melanogaster group *M0* vs. *Mfree*
Gr39a	12	0.19	-	-	-
Gr39aA	10	0.24	NS	-	-
Gr39aB	12	0.14	P<0.011	P<0.0001(*ω*Dgri>1)	P<0.05 (*ω*<1)
				P<0.003 (Dvir+Dmoj*ω*>1)	
Gr39aC	8	0.19	NS	-	P<0.016 (*ω*Dsec = 1.4)
Gr39aD	12	0.18	NS	-	P<0.05 (*ω*<1)
Gr39aE	6	0.27	NS	-	-

*N*, number of sequences tested

*ωM0*, estimates of the overall ratio (*ω*) of nonsynonymous substitution rate to the synonymous substitution rate

NS–not significant

Species abbreviation: Dgri–*D. grimshawii*; Dvir–*D. virilis*; Dmoj–*D. mojavensis*; Dsec–*D. sechellia*

Evidence of expression of *Gr39a* in *D. melanogaster* [6], strong selective constraints, and preservation of the transmembrane domain structure and splice signals all suggest that *Gr39a* is a functionally active gene. The estimates of the coefficient of functional divergence (*θ)* [15]–[17] between the protein isoforms of *Gr39a* are generally high (0.4936–0.6432) (Table 2) and comparable with estimates of *θ* for highly functionally diverged duplicated genes [15] and duplicated *Grs* in Drosophila species (Table S2). These results suggest functional divergence between exons *B, C, D,* and *E*. Interestingly, when we analysed functional divergence between isoforms *B* and *A*, including the single copies of *A* from each species that were considered to be conserved orthologs of *A* of *D. melanogaster*, we detected very low values of *θ* between *A* and *B* (*θ* = 0.1408, not significantly different from zero), providing no evidence that these exons have diverged in their function, but rather suggesting that replacement mutations are due to neutral evolution. However, the inclusion of the duplicate isoforms of *A* of *D. mojavensis*, *D. virilis* and *D. grimshawii* increased *θ* to 0.28–0.38 (P<0.0001), when the *A* and *B* clusters were compared. The comparison of the *A* isoforms of *Sophophora* versus the *A* isoforms of *D. mojavensis, D. virilis* and *D. grimsshawii* also provided evidence of functional divergence between them (*θ* ranged from 0.27 to 0.49, P<0.004–0.002). We also observed low divergence between exons *C* and *D*, possibly indicating similar functions.

**Table 2:** Estimates of functional divergence between the large exons (*A*, *B, C, D, E*) of the *Gr39a* gene.

	A/B	A/E	A/C	A/D	B/E	B/C	B/D	E/C	E/D	C/D
*θ*	0.1408	0.6352	0.5360	0.6432	0.5575	0.5312	0.5944	0.5235	0.4936	0.2928
*α*	2.1806	3.4014	2.9970	2.6127	1.9484	1.6205	1.6260	2.8306	2.3156	2.0193
SE *θ*	0.0873	0.1439	0.1348	0.1016	0.1097	0.1073	0.0812	0.1677	0.0984	0.1091
LR *θ*	2.5998	19.460	15.795	40.038	25.790	24.479	53.458	9.7441	25.144	7.2000
P	NS	0.0001	0.0001	0.0001	0.0001	0.0001	0.0001	0.002	0.0001	0.007

*θ*-maximum likelihood estimate for the coefficient of functional divergence

*α-*maximum likelihood estimate for the gamma shape parameter for rate variation among sites

SE *θ-*standard error of the estimate of *θ*

LR *θ*-likelihood-ratio statistic for comparison of alternative hypothesis *θ*>0 against the null hypothesis of *θ* = 0

P–probability (NS–not significant)

**Figure 4:**
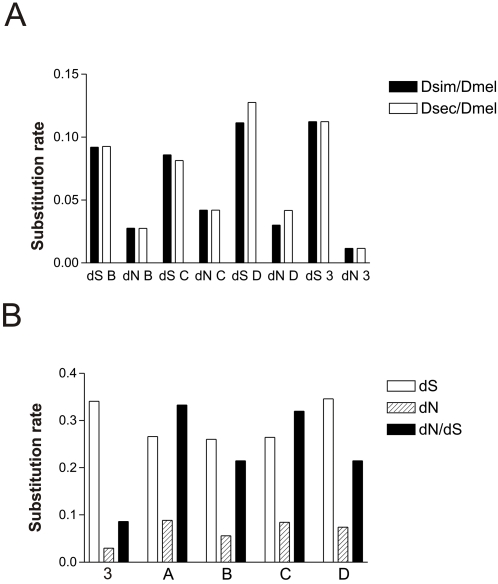
Comparisons of substitution rates and *ω*. A. Pairwise comparison of the *d_S_* and *d_N_* rates of exons *B, C, D* and three conservative small exons (symbol 3 on the figure) in *D. simulans* vs. *D. melanogaster* and *D. sechellia* vs. *D. melanogaster*. B. Results of pairwise comparison of the *d_S_, d_N_* rates and of the *ω* ratio of the exons *A, B, C, D* and three conservative small exons (symbol 3 on the figure) in the pair of species: *D. melanogaster* vs. *D. yakuba*.

We tested the main regions of the *Gr39a* gene for signs of positive selection using several models in PAML. Application of the site and branch-site models detected positive selection on exon *B* (P<0.011) in the subgenus *Drosophila* during the diversification of *D. grimshawi*, *D. mojavensis* and *D. virilis* (Table 1). Only 1.2% of sites of exon *B* underwent positive selection with *ω* = 2.4; sites *28T, 60T, 68S* and *193D*. We applied branch models to test variation in *ω* in exons *B, C* and *D* on phylogenetic lineages within the melanogaster group (we excluded exon *A*, because it degraded in two melanogaster group species). *ω* exceeded 1 (the neutral expectation) in exon *C* of *D. sechellia* (*ω* = 1.43). Analyzing exon *C*, we also found an increase in *ω* in *D. simulans* (*ω = *0.8138) and *D. melanogaster* (*ω = *0.7554). To examine this further, pairwise comparisons of the *d_N_* (nonsynonymous substitution rate) and *d_S_* (synonymous substitution rate) of the exons *C, B, D* (excluding exon *A*, because it degraded in *D. sechellia*) and small conserved exons were carried out in *D. sechellia* vs. *D. melanogaster,* and *D. simulans* vs. *D. melanogaster* (Figure 4A) (comparing closely related species excludes potential problems of accounting for multiple hits at the synonymous sites and saturation). At the conserved small exons the *d_S_* rates exceeded the *d_N_* rates approximately ten times, indicating very strong negative selection against nonsynonymous mutations (Figure 4A). We found signs of a slight increase in the *d_S_* and *d_N_* rates in exon *D* of *D. sechellia*, and also an increase in the *d_N_* rates of exon *C* in *D. sechellia* and *D. simulans*, while the *d_S_* rates of exon *C* were relatively stable compared with the *d_S_* rates in exons *B* or *D*. Thus, the *d_S_* rates of exons *B* and *D* were nearly three times higher than their *d_N_* rates, while in the exon *C* the substitution rate at the synonymous sites was about twice higher than that at the nonsynonymous sites (Figure 4A). For comparison, we also analysed the *d_S_, d_N_* rates and the *ω* ratio *(d_N_/d_S_)* amongst the exons *A, B, C, D* and small conserved exons between *D. melanogaster* and *D. yakuba* (Figure 4B). Along with the increase of the *ω* in the exon *C*, we found similar patterns in exon *A*, in both cases changes of *ω* happened due to an increased rate of nonsynonymous substitution.

## Discussion

About 10% of Drosophila genes contain tandemly duplicated exons, many of which are believed to be involved in mutually exclusive alternative splicing events [18]. Of the chemosensory receptor gene family, which contains around 120 genes, only three gustatory (*Gr23a, Gr28b, Gr39a*) and two olfactory (*Or46a, Or69a*) receptor genes have tandemly duplicated exons, which have been shown to undergo alternative splicing [1]. Among these genes, *Gr39a* is a unique case of rapid evolution through exon duplication and divergence, and in some cases exon loss.

Several structural changes have occurred to *Gr39a*, including the loss of exon *C* in *D. willistoni* and the species of *Drosophila* subgenus, and multiple duplications of exon *A* in *D. pseudoobscura, D. persimilis, D. willistoni, D. mojavensis, D. virilis* and *D. grimshawii*, around the time of the *Drosophila-Sophophora* division. The species of the melanogaster group have lost the ancestral exon *E*, also pseudogenisation of exon *A* has occurred in *D. sechellia* and *D. erecta*. McBride and Arguello [19] also reported pseudogenisation of parts of the *Gr* repertoire in these last two species and related this to the ecological specialisation shown by these species [7]. Evidence for strong purifying constraints on *Gr39a* supports its status as a functional gene. We found evidence of functional divergence between exons *B, E, C* and *D*. However, based on the low level of functional divergence between the exons *C* and *D*, it is likely that they have similar functions, even though both exons have persisted over a long evolutionary period. In *D. melanogaster*, both exons are expressed in the labellum and thorax, but also show spatial delimition with Gr39aC being expressed in the abdomen and Gr39aD in the wings [6]. The increase of the nonsynonymous substitution rate we detected in *Gr39aC* of *D. simulans* and, especially, in the specialist *D. sechellia* must indicate exon diversification.

We found little evidence of functional divergence between exons *A* and *B*. Curiously, exon *A* is multiply duplicated in all lineages except the melanogaster group, where it was lost in the two specialist species. The duplicates of exon *A* present in *D. mojavensis, D. virilis* and *D. grimshawii* show evidence of functional divergence from exon *B*, as well as from exon *A* of the *Sophophora* subgenus. We also detected positive selection acting on *Gr39aB* in the *Drosophila* subgenus. The extensive creation of new copies of exon *A* and signs of positive selection acting on *Gr39aB* in the *Drosophila* subgenus could indicate functional diversification of the *Gr39aA* and *Gr39aB* in these species.

Increased functional diversity results from extensive whole gene duplication in some cases, creating gene families, whereas in other cases it evolves through alternative splicing. Curiously, although the results of these two processes are similar, they are inversely correlated at the genomic level and it is unclear what conditions lead to one rather than the other [20]. There are several hypotheses proposed to explain the functional diversification of duplicated genes which presumably also apply to exon duplication. According to Ohno's hypothesis, most gene duplicates, being functionally redundant, are eliminated from the genome (nonfunctionalization), except for rare occasions when beneficial mutations could lead to a new gene function (neofunctionalization) [21]. Another model predicts dividing the functions between the duplicate and the original copy (subfunctionalization) [22]. Both models assume that relaxation or a lack of selection on the duplicate allows the acquisition of novel replacement mutations and/or changes in expression pattern. According to Hughes [23], “specialization” of duplicates to different functions of the bi-functional ancestral gene can occur through positive selection. This model assumes a bi-functional nature of the ancestral gene, though in the case of already extensive gene families, different loci will already have similar functions but a degree of specialization (such as detecting related ligands). It has been shown that many chemoreceptors can recognize more than one ligand [24], suggesting their bi- or multi-functional nature. Duplication of such genes can facilitate the specialization of daughter genes and changes in expression, for instance in more restricted sets of tissues, can further drive specialization [23]. For tandemly duplicated exons, Kondrashov and Koonin [25] favour the “specialization” model of the duplicated exons, assuming that if both duplicated exons are translated immediately after duplication, both will be subject to stabilizing selection, which excludes the possibility of short-term relaxation or lack of selection required to allow accumulation of replacement mutations. Alternatively, adaptation of alleles to different functions or sub-functions might start even before duplication [26], thereby facilitating neofunctionalisation after duplication.

Despite our expectations that large alternatively spliced exons of the *Gr39a* would experience similar selective constraints and stabilizing selection, we found evidence of relaxation in the selective constraints on exon *A* (which is prone to duplication) and exon *E*. Exon *E* is either present as a single copy or lost in some lineages, while exon *A* has multiply duplicated in most species. We found evidence of functional divergence between the copies of *A* amongst the species of two subgenera, and we suggest that the exon divergence probably occurred through relaxation of selective constrains on this exon, which implies subfunctionalization of the duplicates according to Lynch and Force [22] or neofunctionalization according to Ohno's hypothesis [21]. The selective constraints that act on other large exons (*B, C* and *D*) are relatively constant. We detected signals of positive selection on the *Gr39aB* in the *Drosophila* subgenus and possibly on *Gr39aC* in some species of the melanogaster group. Exon *B* is present as a single copy in all Drosophila species examined; the signature of positive selection which we detected on this exon indicates an adaptive mode of its evolution. The ancient duplicates *Gr39aC* and *Gr39aD* have functionally diverged, but experience similar selective constrains. We suggest the participation of positive selection in their divergence. We found some evidence of an increase of the nonsynonymous substitution rate in several species of the melanogaster group with the strongest signal in the specialist *D. sechellia*. This observation might support Hughes's model of “specialization” of duplicates [23] or the idea that positive selection can lead to a completely new function (neofunctionalization).

## Materials and Methods

Annotation of the orthologs of *D. melanogaster Gr39a* genes was performed for the other 11 Drosophila species whose genome sequences are publicly available [10] using a combination of Blast [27], GeneWise [28] and manual curation [7]. Annotations (Table S1, Figure S1) and proteins alignment (Figure S2) are available as Supplemental data. Figure S1 contains sequences of genes from the start codon to stop including introns, and also contains coding sequences (open reading frames, ORFs) in which introns are represented as gaps. The resulting sets of *Gr39a* sequences were multiply aligned at the codon level using ClustalW [29] on translations, followed by Protal2dna (K. Schuerer, C. Letondal; http://bioweb.pasteur.fr). Coding sequences identified were tested for the presence of transmembrane domains in their product using the TMHMM2.0 program [30]. For comparison of the *Gr39a* locus with other Drosophila gustatory (*Gr23a, Gr28b*, *Gr39a)* and olfactory (*Or46a, Or69a*) receptor genes that are known to undergo alternative splicing, we also identified their orthologs in the same set of species following the same procedure.

The phylogeny of *Gr39a* nucleotide sequences, with *Gr68aDmel* and *Gr32aDmel* as outgroups, was reconstructed by Bayesian Markov chain Monte Carlo (MCMC) analysis using MrBayes-3.1.2 [31] with the HKY+4Γ model; default priors on branch lengths, rate parameters and tree topology; and two runs, each with one chain of 30,000,000 generations sampled every 50,000 generations. The first 250 trees sampled in each run were discarded as burn-in, leaving a final MCMC sample of 702 trees. Convergence was assessed using the average standard deviation of split frequencies, output by MrBayes. Independence within the sample was assessed using autocorrelation in tree log-likelihoods of the 351 trees from each run [32], obtained with the ACF function of Minitab v14.20 (Minitab, inc.). The majority-rule consensus of the final MCMC sample was taken to be the phylogeny of *Gr39a*.

We used Notung 2.1 [14] to reconcile the “gene” tree (in our case, exon tree) and the species tree. Notung maps duplication and loss events onto branches of the species tree by reconstructing ancestral states according to parsimony rules. The topology of the species tree (Figure 1A) was obtained from [10].

The structural and potentially functional divergence of the large exons of *Gr39a* was explored using DIVERGE v1.04 [15]. The approach applied in DIVERGE was developed for the analysis of the functional divergence of duplicated genes which, from an evolutionary perspective, we assume also applies to duplicated exons. This approach was also suggested to be useful for studying functional divergence after speciation events, domain shuffling, lateral gene transfer etc. [15]. Briefly, after gene (or exon) duplication, the evolutionary rate (*λ*) at an amino acid site may increase, leading to a functional divergence in the early stage after duplication, followed later by purifying constraints acting to maintain the novel function(s) [15]. If the evolutionary rates between the original (*λ_1_*) and duplicate (*λ_2_*) stay the same or change proportionally over time, the coefficient of rate correlation (*r_λ_*) between them will be 1. A decrease of the *r_λ_* indicates differences in the evolutionary rates between the original and the duplicate copy, and a measure of such divergence is assigned as *θ = 1−r_λ_*, where *θ* is a coefficient of functional divergence [15]. *θ* = 0 indicates no functional divergence, and an increase in *θ* from 0 to 1 shows increasing functional divergence from weak to extremely strong [15]. The significance of *θ* is assessed using the likelihood ratio statistic, *LR*, defined as 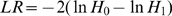 where *H*_0_ is the likelihood of the model representing the null hypothesis (here, a model in which *θ* is constrained to equal zero) and *H*_1_ is the likelihood of a more general model, representing the alternative hypothesis (*θ* is allowed to vary). *LR* was converted to a P-value on the assumption that the null distribution of *LR* is χ^2^ with degrees of freedom (d.f.) equal to the difference in the number of free parameters between *H*_1_ and *H*_0_ (here, d.f. = 1) [15], [33]. We estimated *θ* between the encoded protein isoforms Gr39aA, Gr39aB, Gr39aC, Gr39aD and Gr39aE, excluding the last transmembrane domain specified by three conserved exons. Because exon *A* duplicated in most lineages, we performed several comparisons of the Gr39aA isoforms. Initially, we compared the Gr39aA isoforms with Gr39aB, Gr39aC, Gr39aD and Gr39aE, by including only single copies of *A* from each species that were considered to be conserved orthologues to the *A* of *D. melanogaster* on the basis of the highest similarity score calculated by GeneWise, then repeated this test by excluding *D. grimshawi* whose copies of A scored similar values in GeneWise. We then repeated the analysis including duplicates of Gr39aA of one of the species where exon A duplicated (*D. pseudoobscura*, *D. persimilis*, *D. willistoni, D. mojavensis*, *D. virilis* and *D. grimshawi*) and compared these with Gr39aB. We also compared the Gr39aA of the *Drosophila* subgenus with the Gr39aA isoforms of the *Sophophora* subgenus.

The “M0” model of *codeml* in the PAML computer package [34] was used to determine the average selective constraint on the whole gene, and also separately for its exons (*B, C, D, E* and the set of exons *A*, which comprised from the copies of *A* that were considered to be conserved orthologues to the *A* of *D. melanogaster*), through estimation of the ratio of the normalized nonsynonymous substitution rate (*d_N_*) to normalized synonymous substitution rate (*d_S_*), or *ω = d_N_/d_S_*. *ω*>1 is considered strong evidence of positive selection for amino acid replacements whereas *ω*≈0 indicates purifying selection, while *ω* = 1 conforms to a neutral expectation [35]. To test for positive selection on orthologous exons, we used a range of tests in which two models are compared, again by means of *LR*. The models permit detecting episodes of positive selection acting on a fraction of sites (“site” models) or on particular phylogenetic lineages (“branch” models), as well as a combination of both (“branch-site” models). The strength of evidence for site, branch and branch-site models was assessed by comparing *codeml* models “M8” with “M7”, “Mfree” with “M0”, and “M2” with “M1”, respectively. Model M7 allows several site classes with *ω* drawn from a *β* distribution and fixed between 0 and 1, while M8 adds another class of sites with *ω*>1 [34]. Branch models allow testing variation in *ω* among different branches of a phylogeny [34]. The simplest model, M0, assumes one *ω* for all branches, while the “free-ratio” or Mfree model allows different *ω* for all branches [34]. We used M0 and Mfree to estimate *ω* for the exons *B, C* and *D* in the melanogaster group (we excluded exon *A* because it had degraded in two species of the melanogaster group). Finally, branch-site models can detect episodic events of adaptive evolution on specific sites in different branches of a phylogeny [34]. In the M1 model, there are four classes of sites with ω fixed below 1, while M2 allows a fraction of sites to have *ω*>1 on user-selected (“foreground”) branches [34]. For converting *LR* to a P-value, for M8 vs M7, d.f. = 2; for Mfree vs M0 in this study, d.f. = 8; for M1 vs M0, d.f. = 1. Finally, the pairwise comparison of the *d_S_* and *d_N_* rates was performed using *codeml* option runmode = −2 [34].

## Supporting Information

Table S1 Annotation and description of the identified Gr39a genes
(0.02 MB XLS)

Table S2 The estimates of the functional divergence amongst Drosophila gustatory receptors
(0.02 MB XLS)

Figure S1 The nucleotide sequences of the identified Gr39a genes
(0.23 MB TXT)

Figure S2 The Gr39a multiple protein sequence alignment
(10.06 MB TIF)
