# Supplementary figures and images for: Evolution of a Complex Locus: Exon Gain, Loss and Divergence at the Gr39a Locus in Drosophila
Source: PLoS One. 2008 Jan 30;3(1):e1513. doi: 10.1371/journal.pone.0001513 (PMC2204066; doi:10.1371/journal.pone.0001513)

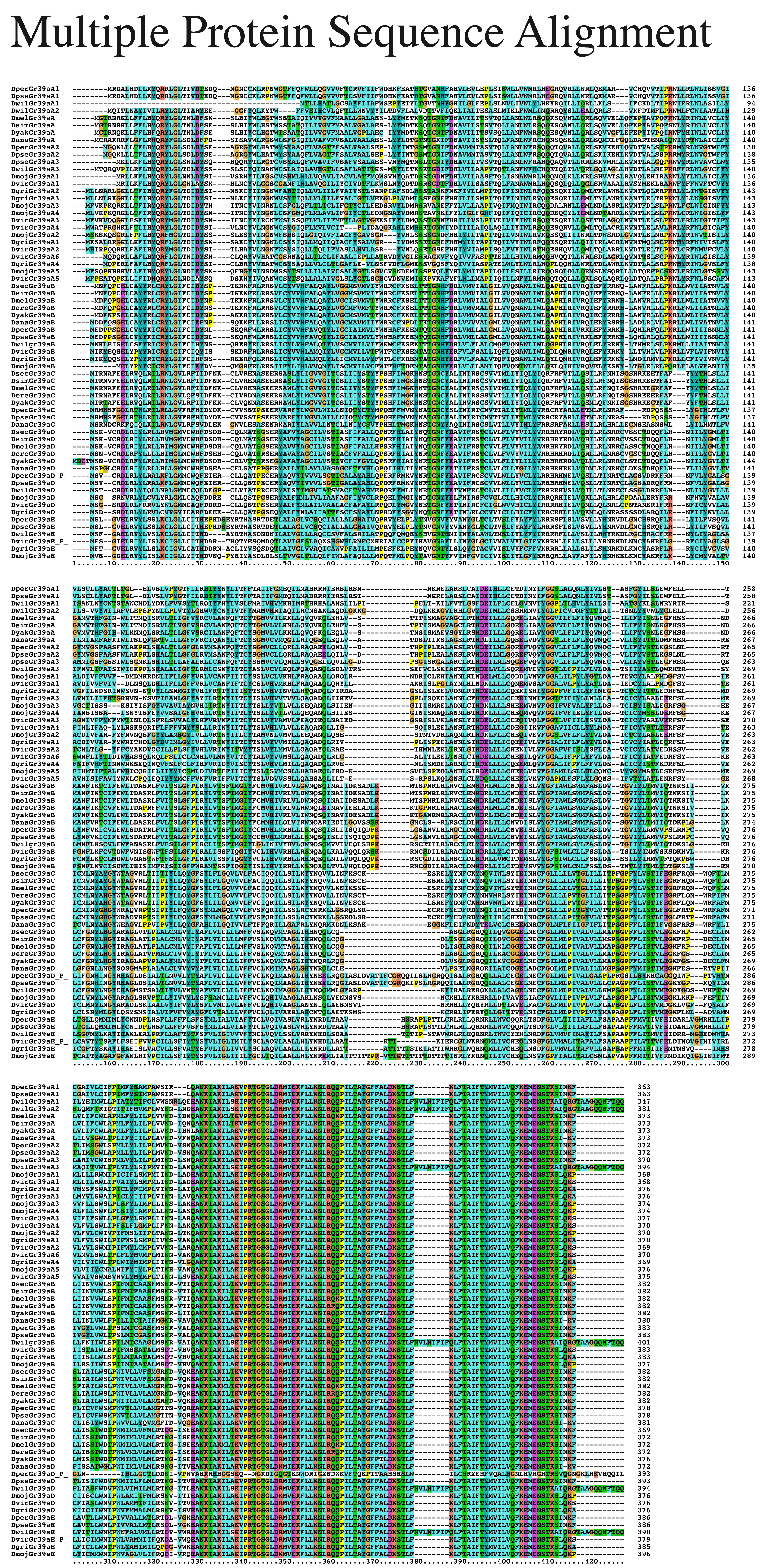

Supplement: Figure S2 — The Gr39a multiple protein sequence alignment (10.06 MB TIF) [file pone.0001513.s004.tif]
